# Premature ejaculation

**DOI:** 10.4103/0970-1591.32056

**Published:** 2007

**Authors:** Chris G. McMahon

**Affiliations:** Australian Centre For Sexual Health Suite 2-4, Berry Road Medical Centre 1a Berry Rd St. Leonards, Australia

**Keywords:** Premature ejaculation; selective serotonin reuptake inhibitors, dapoxetine, intravaginal ejaculatory latency time, PDE-5 inhibitors

## Abstract

Premature ejaculation (PE) is a common male sexual disorder. Recent normative data suggests that men with an intravaginal ejaculatory latency time (IELT) of less than 1 minute have “definite” PE, while men with IELTs between 1 and 1.5 minutes have “probable” PE. Although there is insufficient empirical evidence to identify the etiology of PE, there is limited correlational evidence to suggest that men with PE have high levels of sexual anxiety and inherited altered sensitivity of central 5-HT (5-hydroxytryptamine, serotonin) receptors. Pharmacological modulation of the ejaculatory threshold using off-label daily or on-demand selective serotonin re-uptake inhibitors is well tolerated and offers patients a high likelihood of achieving improved ejaculatory control within a few days of initiating treatment, consequential improvements in sexual desire and other sexual domains. Investigational drugs such as the ejaculo-selective serotonin transport inhibitor, dapoxetine represent a major development in sexual medicine. These drugs offer patients the convenience of on-demand dosing, significant improvements in IELT, ejaculatory control and sexual satisfaction with minimal adverse effects.

Premature ejaculation (PE) is one of the most common male sexual disorders and has been estimated to occur in 4-39% of men in the general community.[1–7] The World Health Organization (WHO) 2^nd^ International Consultation on Sexual Health defined it as “… persistent or recurrent ejaculation with minimal stimulation before, on or shortly after penetration and before the person wishes it, over which the sufferer has little or no voluntary control which causes the sufferer and / or his partner bother or distress…”[8] This multivariate definition encompasses the main dimensions of PE - ejaculatory latency, control and sexual satisfaction.

Most community-based epidemiological studies are limited by their reliance on either patients' self-reports of PE or inconsistent and poorly validated definitions of PE. A recent multinational community-based age-ranging study of an unselected “normal” population of 500 heterosexual couples involving stopwatch timing of the intravaginal ejaculatory latency time (IELT) during sexual intercourse, has provided previously lacking normative data.[9] This study demonstrated that the distribution of the IELT was positively skewed, with a median IELT of 5.4 minutes (range, 0.55-44.1 minutes) [Figure 1]. The median IELT decreased with age and varied between countries. The authors regarded the 0.5 and 2.5 percentiles as acceptable standards of disease definition in this type of skewed distribution. They proposed that men with an IELT of less than 1 minute (belonging to the 0.5 percentile) have “definite” premature ejaculation, while men with IELTs between 1 and 1.5 minutes (between 0.5 and 2.5 percentiles) have “probable” PE.[10]

**Figure 1:**
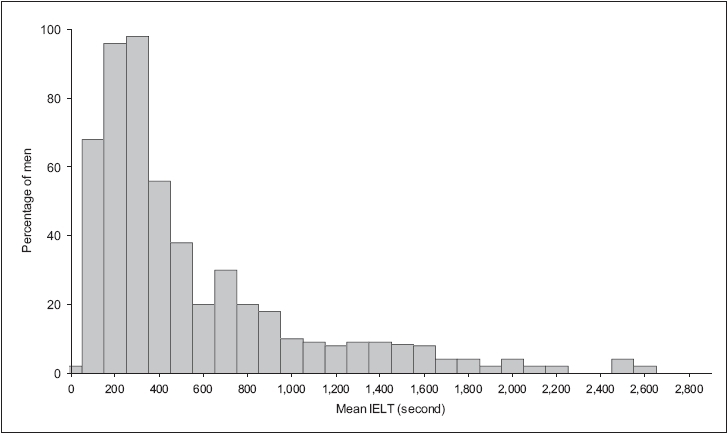
Distribution of intravaginal ejaculatory latency times values in a random cohort of 491 men[60]

There is little published data on the impact of birth country, religion or culture on the prevalence of PE. An increased susceptibility to premature ejaculation in men from the Indian subcontinent has been reported.[11,12] Kinsey's observation that Asian men have shorter times to ejaculation than Caucasians, who in turn have shorter times to ejaculation than Afro-Caribbeans, has been interpreted to suggest that some races are more “sexually restrained” than others.[13,14] A recent study reported a preponderance of men from Middle Eastern and Asian backgrounds presenting for treatment of PE which exceeded the representation of these ethnic groups in the local population.[15,16]

The premise that premature ejaculation is a psychosomatic disturbance due to a psychologically overanxious personality was first suggested by Schapiro in 1943. He classified PE as primary (lifelong) or secondary (acquired).[17] The behavioristic view that chronic PE was the result of performance anxiety related to a disturbing initial episode of premature ejaculation was first proposed by Masters and Johnson.[18] Most of the behavioural treatments currently used are based on this premise.

In a study of 1326 consecutive men with PE, lifelong PE was present in 736 men (74.4%) and acquired PE was present in 253 men (25.6%).[19] Men with PE appear younger than those without and after adjusting for concomitant erectile dysfunction (ED), the risk of PE was found to significantly decrease with aging.[20] Higher levels of education, divorce and the presence of social phobia appear to increase the risk of PE.[20,21] A decreased risk of PE has been reported in men with treated diabetes, while no association was found with hypertension, cardiac disease, hypercholesterolemia and peripheral or central neuropathy. Men with self-reported PE have a lower frequency of sexual intercourse, higher levels of intercourse-related anxiety and note greater impairment in intercourse satisfaction and sexual relationship satisfaction compared to men without PE.[22] However, they do not report a reduced quality of life, reduced sexual desire or a reduced ability to become sexually aroused.[8,22]

Over the past 15 years, an increasing number of publications have reported pharmacological treatment of PE with a variety of different medications, which act centrally or locally to retard the psychoneurological control of ejaculation and subsequent orgasm.[23] It is well established that major tranquillisers and selective serotonin re-uptake inhibitor drugs (SSRIs) retard ejaculation significantly and will, in a small percentage of men, result in anejaculation.[24–26] The efficacy of SSRIs in delaying ejaculation combined with their low side effect profile make them first-line therapeutic agents for premature ejaculation administered either on a daily or an “on-demand basis”.[27,28]

## PHYSIOLOGY OF EJACULATION

Ejaculation is a reflex comprised of sensory receptors and areas, afferent pathways, cerebral sensory areas, cerebral motor centres, spinal motor centres and efferent pathways. There are three basic mechanisms involved in normal antegrade ejaculation-emission, ejection and orgasm.[29] Emission is the result of a sympathetic spinal cord reflex initiated by genital and / or cerebral erotic stimuli and involves the sequential contraction of accessory sexual organs. Considerable initial voluntary control of emission progressively decreases until the point of ejaculatory inevitability.[30] Ejection also involves a sympathetic spinal cord reflex upon which there is little or no voluntary control. Ejection involves bladder neck closure, rhythmic contractions of bulbocavernous, bulbospongiosus and other pelvic floor muscles and relaxation of the external urinary sphincter.[30] Orgasm is the result of cerebral processing of pudendal nerve sensory stimuli resulting from increased pressure in the posterior urethra, sensory stimuli arising from the veramontanum and contraction of the urethral bulb and accessory sexual organs.

**Figure 2:**
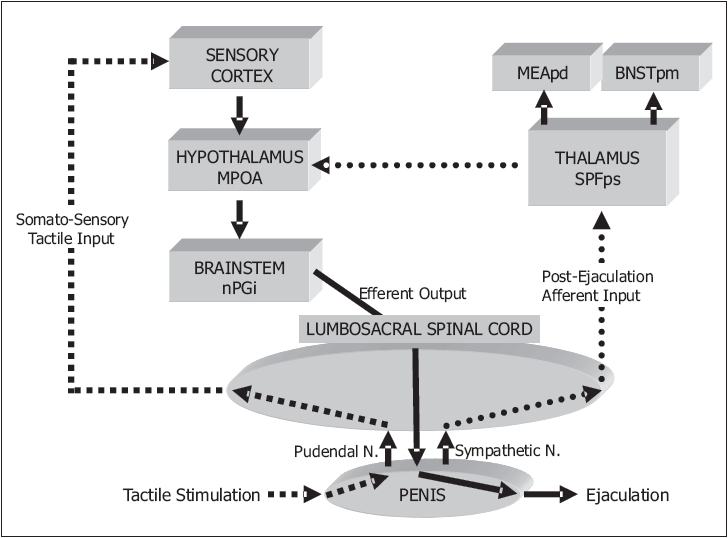
Central nervous system areas involved before, during and after ejaculation. Somatosensory tactile input form the penis / genitals ascends to the cerebral cortex. Efferent pathways project from the hypothalamus to the sacral spinal cord and genitals. After ejaculation, information is returned from the genitals to several brain areas. MEApd: Posterodorsal medial amygdala, BNSTpm: Posteromedial bed nucleus of stria terminalis, MPOA: medial preoptic area, nPGi: nucleus paragigantocellularis, SPFps: medial parvicellular subparafascicular nucleus of thalamus[60]

The ejaculatory reflex is predominantly controlled by a complex interplay between central serotonergic and dopaminergic neurons with secondary involvement of cholinergic, adrenergic, nitrergic, oxytocinergic, galanergic and GABAergic neurons. The cerebral events which occur during ejaculation and the abnormalities present in men with PE have not been clearly defined with positron emission tomography (PET) and functional magnetic resonance imaging (fMRI) techniques. Seminal emission and ejection are integrated into the complex pattern of copulatory behavior by several forebrain structures including the medial preoptic area (MPOA) and the nucleus paragigantocellularis (nPGi) [Figure 2].[31,32] Descending serotonergic pathways from the nPGI to the lumbosacral motor nuclei tonically inhibit ejaculation.[32] Disinhibition of the nPGI by the MPOA facilitates ejaculation. A population of lumbar spinothalamic neurons has been identified in male rats (LSt cells) that constitute an integral part of the generation of ejaculation. LSt cells send projections to the autonomic nuclei and motoneurons involved in the emission and expulsion phase and receive sensory projections from the pelvis.[33] Several brain areas are activated after ejaculation by ascending fibres from the spinal cord and may have a possible role in satiety and the postejaculatory refractory time.

Animal and human sexual psychopharmacological studies have attributed a serotonergic basis and possible genetic etiology to premature ejaculation.[34–37] Male rat studies demonstrate that serotonin and 5-HT receptors are involved in the ejaculatory process. The speed of ejaculation appears to be determined by 5-HT2C and 5-HT1A receptors. Stimulation of 5-HT2C receptors with non-selective 5-HT2C agonists delays ejaculation in male rats whereas stimulation of postsynaptic 5-HT1A receptors resulted in shorter ejaculation latency.[38] Administration of SSRIs results in active blockade of presynaptic membrane 5-HT transporters and the resultant higher synaptic cleft levels of 5-HT activate post-synaptic 5-HT2C and 5-HT1A receptors to delay ejaculation.[35,39]

## DEFINING PREMATURE EJACULATION

Medical literature contains several univariate and multivariate operational definitions of PE. The lack of agreement as to what constitutes PE has hampered basic and clinical research into the etiology and management of this condition. Quantitative measures of intercourse such as the IELT and subjective patient reported outcome (PROs) measures of voluntary control over ejaculation or self efficacy such as the extent of sexual satisfaction and the level of bother or distress have been described and employed as patient-related outcomes in premature ejaculation clinical trials. Each of the three criteria above has been operationalized although not always with consistency.[40]

## INTRAVAGINAL EJACULATORY LATENCY TIME (IELT)

Operationalization of PE using the length of time between penetration and ejaculation—the IELT, forms the basis of most current clinical studies on PE. There is considerable variance of the latencies used to identify men with PE with IELTs ranging from 1-7 min and none of the definitions is based on normative data or offers any supportive rationale for their proposed cut-off time for IELT.[41–44] An average duration of intercourse of 4-7 min was reported by Gebhard, suggesting that ejaculation before 4 min after intromission should be considered premature.[45]

Waldinger *et al* reported IELTs of less than 30 sec and less than 60 seconds in 77 and 90% of 110 men with PE respectively.[46] McMahon *et al* reported similar results in 1346 consecutive men with PE and a mean IELT of 43.4 seconds.[19] Predominant anteportal ejaculation (during foreplay) occurred in 5.6% of men. Although normative data is lacking, it is reasonable for clinicians to regard men who ejaculate within 2 minutes of penetration as suffering from PE. Anteportal ejaculation or ejaculation within 1 minute should be regarded as severe PE.

### Sexual satisfaction

Men with PE report lower levels of sexual satisfaction compared to men with normal ejaculatory latency. Patrick *et al* reported ratings of “very poor” or “poor” for sexual satisfaction in 31% of men with PE compared to 1% in a group of normal controls.[47] The inability to control and defer ejaculation until the female partner was sexually satisfied in at least 50% of intercourse attempts was proposed as a definition of PE by Masters and Johnson.[48] An inherent problem exists in defining a man as dysfunctional based on the sexual responsiveness of his partner. This definition implies that any male whose female partner has difficulty in reaching orgasm should be labeled as a premature ejaculator. This definition is at odds with the report that only 30% of women achieve orgasm during sexual intercourse regardless of the extent of their partner's ejaculatory control and latency. Rowland reported that over 89.4% of men with self-reported PE regarded fulfilling their partner's sexual needs as very or extremely important.[49]

### Voluntary control

Kaplan and other authors have suggested that an inability to voluntarily defer ejaculation defines PE.[50–53] This definition has yet to be adequately operationalized to allow comparison across subjects or across studies. Grenier and Byers failed to demonstrate a strong correlation between ejaculatory latency and subjective ejaculatory control.[4] They reported that some men with a brief ejaculatory latency time reported adequate ejaculatory control and vice versa and concluded that the dimensions of ejaculatory control and latency are distinct concepts. Contrary to this, other authors have reported a moderate correlation between IELT and the feeling of ejaculatory control.[46,47] Patrick *et al* reported ratings of “very poor” or “poor” for control over ejaculation in 72% of men with PE as compared to 5% in a group of normal controls.[47]

## DISTRESS

Existing definitions of PE include distress as an important dimension of PE.[8,23,54] However, the word distress has negative social implications and its existence is denied by most men with PE. This dimension of PE is better captured by the word “bother”. The extent of bother defines the severity of PE. One study reported that 64% of men with PE rated their extent of personal distress as “quite a bit” or “extremely” compared to 4% in a group of normal controls.[47]

Although partner distress is a significant contributor to treatment-seeking behavior, there is limited information regarding the effect of PE on the partner. Several studies have reported that the effects of PE on the female partner are integral to understanding the impact of PE on the male and on the sexual relationship as a whole.[55–57] Patrick *et al*. reported that 44% of partners of men with PE rated their extent of personal distress as “quite a bit” or “extremely” compared to 3% in a group of partners of normal controls.[47] Patrick *et al* also reported that partner PRO measures differentiated men with PE from men without PE and correlated moderately with measures of IELT and subject PRO measures. However, partner perceptions of PE generally indicated less dysfunction than those of subjects.[47] Although PE adversely affects partner sexual satisfaction, it appears to have minimal impact upon relationship satisfaction.[56] Furthermore, partners of men with PE report relatively high levels of female sexual dysfunction.[58,59] The observation that PE often predates the time of onset of the women's sexual symptoms suggests that PE may be a risk factor for female sexual dysfunction.[58]

The design of all future studies on any aspect of PE should include a uniform operationalized multivariate definition of PE where the dimensions of latency, control, satisfaction and distress / bother are defined, measured and analysed as continuous variables without arbitrary cut-off values.

## THE ETIOLOGY OF PREMATURE EJACULATION

Historically, attempts to explain the etiology of PE has included a diverse range of biological and psychological theories. Most of these proposed etiologies are not evidence-based and are speculative at best. Psychological theories include the effect of early experience and sexual conditioning, anxiety, sexual technique, the frequency of sexual activity and psychodynamic explanations. Biological explanations include evolutionary theories, penile hypersensitivity, central neurotransmitter levels and receptor sensitivity, degree of arousability, the speed of the ejaculatory reflex and the level of sex hormones.

There is little empirical evidence to suggest a causal link between PE and any of the factors thought to cause PE. There is, however, limited correlational evidence to suggest that lifelong PE is a genetically determined biological variable related to the inherited sensitivity of central 5-HT receptors whereas acquired PE is due to high levels of sexual anxiety, ED or lower urinary tract infection.

Ejaculatory latency time is probably a biological variable, which is genetically determined and may differ between populations and cultures, ranging from extremely rapid through average to slow ejaculation. Hyposensitivity of the 5-HT2C and / or hypersensitivity of the 5-HT1A receptors have been suggested as a possible explanation of lifelong PE.[39,60] Men with low 5-HT neurotransmission and probable 5-HT2C receptor hyposensitivity may have their ejaculatory threshold genetically “set” at a lower point and ejaculate quickly and with minimal stimulation. On the other hand, men with a higher set-point can sustain more prolonged and higher levels of sexual stimulation and can exert more control over ejaculation. Men with a very high set-point may experience delayed or absent ejaculation despite achieving a full erection and prolonged sexual stimulation. Treatment with an SSRI class drug activates the 5-HT2C receptor, elevates the ejaculatory threshold set-point and delays ejaculation. The extent of ejaculatory delay may vary widely in different men according to the dosage and frequency of administration of SSRI and the genetically determined ejaculatory threshold set-point. Cessation of treatment results in re-establishment of the previous set-point within 5-7 days in men with lifelong PE.

Anxiety has been reported as a cause of PE by multiple authors and is entrenched in the folklore of sexual medicine as its most likely cause despite scant empirical research evidence to support any causal role.[17,50,61] Several authors have suggested that anxiety activates the sympathetic nervous system and reduces the ejaculatory threshold as a result of an earlier emission phase of ejaculation. Several authors have suggested the possibility that high levels of anxiety and excessive and controlling concerns about sexual performance and potential sexual failure might distract a man from monitoring his level of arousal and recognising the prodromal sensations that precede ejaculatory inevitability.[52,53,62–65] The causal link between anxiety and PE is speculative, unsupported by any empirical evidence and is in fact, contrary to empirical evidence from some researchers.[66]

Recent data demonstrates that almost half of the men with ED also experience PE.[67] Men with early ED may intentionally “rush” sexual intercourse to prevent premature loss of their erection and ejaculate with a brief latency. This may be compounded by the presence of high levels of performance anxiety related to their ED which serves to only worsen their prematurity. In the absence of a thorough sexual history, these men may be incorrectly diagnosed as suffering from PE and not the underlying ED.

## PE DRUG TRIAL DESIGN

The results of PE drug clinical trials are only reliable, interpretable and capable of being generalised to patients with the disorder studied when conducted in well defined and consistent populations, differentiation of lifelong and acquired PE as separate PE subgroups, exclusion or treatment as a separate subgroup subjects with erectile dysfunction (ED) or other co-morbid sexual disorders, using a double-blind placebo controlled study design, and consistent objective physiological measures or sensitive, validated outcome assessment instruments as study endpoints.[68] In PE studies, the study population should be well characterised, representative of the overall patient population and defined using a multivariate definition of PE. As the population of men with PE is not homogenous, lifelong and acquired PE should be treated as demographically and etiologically distinct disorders and analysed as separate PE subgroups.[19] Subjects should be involved in a stable, monogamous heterosexual relationship, prepared to attempt intercourse on a regular basis and provide written informed consent. The presence of comorbid ED should be evaluated using a validated instrument such as the International Index of Erectile Function (IIEF) and patients with any degree of ED should be either excluded from the study or treated as a separate subgroup. Patients with hypoactive sexual desire or other sexual disorders, urogenital infection, major psychiatric disorders, a history of drug and alcohol abuse or contraindications to the study drug should be excluded from the study.Measurement of the intravaginal ejaculatory latency time (IELT) by stopwatch is the best method to diagnose PE and the response to treatment and should be used as a primary efficacy endpoint. Laboratory studies of ejaculatory dysfunction may be simplified by the use of the Sexual Assessment Monitor (SAM), an electronic data collector which comprises a vibrator to induce ejaculation and a sensor to measure time-to-erection and IELT by the detection of ejaculatory pulses, but the role of such devices in large at-home Phase III clinical trials is limited.[69] Recent normative IELT data supports earlier suggestions by several authors that intravaginal ejaculatory latency times (IELTs) of less than 1 minute or less than 2 minutes be regarded as cut-points for inclusion in a clinical trial.[9,27,40] Subjective patient reported outcomes (PROs) of ejaculatory control, sexual satisfaction and bother/distress are important additional efficacy endpoints and can be evaluated using validated patient reported outcome instruments.[70–73] Research into the development of validated, reliable and consistent patient reported outcome measures is ongoing.

## TREATMENT

### Psychosexual counselling

In many relationships, PE causes few if any problems. In others, the couple may reach an accommodation of the problem through various strategies-young men with a short refractory period may often experience a second and more controlled ejaculation during a subsequent episode of lovemaking. Frequently however, PE eventually leads to significant relationship problems with partners regarding the man as selfish and developing a pattern of sexual avoidance. This only worsens the severity of the prematurity on the occasions when intercourse does occur.

The cornerstones of behavioural treatment are the Seman's “stop-start” manoeuvre and its modification proposed by Masters and Johnson, the squeeze technique. Both are based on the theory that PE occurs because the man fails to pay sufficient attention to preorgasmic levels of sexual tension.[48,74] As most men with PE are aware of their anxiety, the sources of such anxiety being relatively superficial, treatment success with these behavioural approaches is relatively good in the short term though convincing long-term treatment outcome data is absent.[51,75–77]

### Pharmacological treatment

Pharmacological modulation of ejaculatory threshold represents a novel and refreshing approach to the treatment of PE and a radical departure from the psychosexual model of treatment, previously regarded as the cornerstone of treatment. The introduction of SSRIs has revolutionized the approach to and treatment of PE. SSRIs consist of five compounds citalopram, fluoxetine, fluvoxamine, paroxetine and sertraline with a similar pharmacological mechanism of action. Although the methodology of the initial drug treatment studies was rather poor, later double blind and placebo-controlled studies replicated the genuine effect of clomipramine and SSRIs to delay ejaculation. Inspite of an increasing inclination towards research into more evidence-based drug treatment, the majority of studies still lack adequate design and methodology.[78] A recent meta-analysis of all drug treatment studies demonstrated that only 14.4% had been performed according to the established criteria of evidence-based medicine. Open design studies and studies using subjective reporting or questionnaires showed a higher variability in ejaculation delay than double-blind studies in which the ejaculation delay was prospectively assessed with a stopwatch.[78]

### Daily treatment with selective serotonin reuptake inhibitors

**Figure 3:**
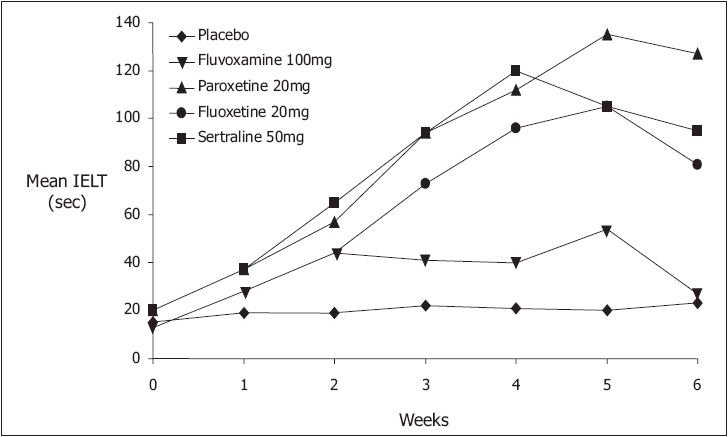
Selective serotonin reuptake inhibitors produce ejaculatory delay within 5-10 days[27]

Paroxetine 20-40 mg, clomipramine 10-50 mg, sertraline 50-100 mg and fluoxetine 20-40 mg can be used for daily treatment [Figure 3]. Paroxetine appears to exert the strongest ejaculation delay, increasing IELT approximately 8.8 fold over baseline.[78] Ejaculation delay usually occurs within 5-10 days but may occur earlier. Adverse effects are usually minor, start in the first week of treatment, gradually disappear within 2-3 weeks and include fatigue, yawning, mild nausea, loose stools or perspiration. Diminished libido or mild ED is not reported frequently. Significant agitation is reported by a small number of patients and treatment with SSRIs should be avoided in men with a history of bipolar depression.

### On-demand treatment with selective serotonin reuptake inhibitors

**Figure 4:**
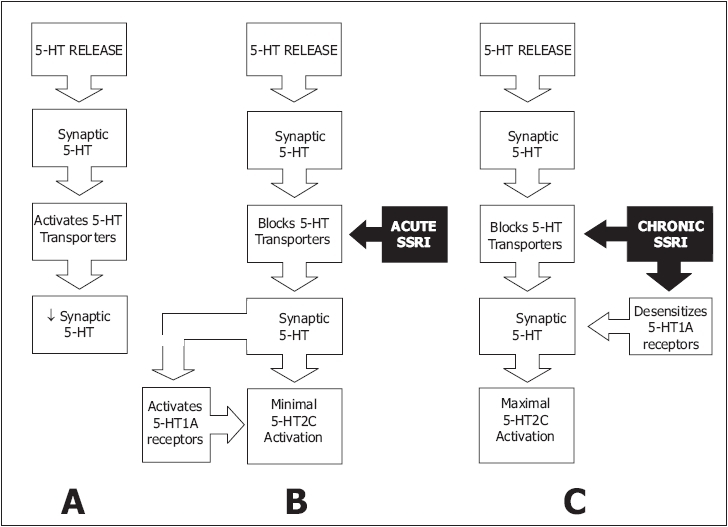
A. Synaptic cleft 5 HT and 5-HT neurotransmission are regulated by somatodendritic 5 HT1A autoreceptors, presynaptic 5 HT1B / 1D autoreceptors and a 5 HT transporter re uptake system. As 5-HT is released into the synaptic cleft from presynaptic axonal vesicles, 5 HT transporters again take up and remove 5 HT from the synaptic cleft, preventing overstimulation of the postsynaptic receptors. B. After blockage of 5 HT transporters by acute administration of selective serotonin re-uptake inhibitor class drugs: SSRIs;, synaptic cleft 5 HT increases but is counteracted by activation of 5 HT1A autoreceptors which inhibit further 5 HT release. C. Chronic administration of SSRIs results in greatly enhanced 5-HT neurotransmission due to several adaptive processes which may include presynaptic 5-HT1A and 5-HT1B / 1D receptor desensitisation[35]

Administration of clomipramine, paroxetine, sertraline, fluoxetine 4-6 hours before intercourse is efficacious and well tolerated but is associated with less ejaculatory delay than with daily treatment. Daily administration of an SSRI is associated with superior fold increases in IELT compared to on-demand administration. This is due to greatly enhanced 5-HT neurotransmission resulting from several adaptive processes which may include presynaptic 5-HT1a and 5-HT1b / 1d receptor desensitisation [Figure 4].[39] On-demand treatment may be combined with either an initial trial of daily treatment or concomitant low dose daily treatment.[28,79,80]

### On-demand treatment with dapoxetine

A number of rapid-acting, short half-life SSRIs are under investigation as on-demand treatments for PE. Dapoxetine is the first compound specifically developed for the treatment of PE. Dapoxetine is a potent SSRI (pK_i_ = 8 nM), which is structurally similar to fluoxetine [Figure 4].[81] Equilibrium radioligand-binding studies using human cells demonstrate that dapoxetine binds to 5-HT, norepinephrine (NE) and dopamine (DA) reuptake transporters and inhibits uptake in the following rank order of potency: NE < 5-HT >> DA.[82] Brain PET studies have demonstrated significant displaceable binding of radiolabeled dapoxetine in the cerebral cortex and subcortical grey matter.[83]

**Figure 5:**
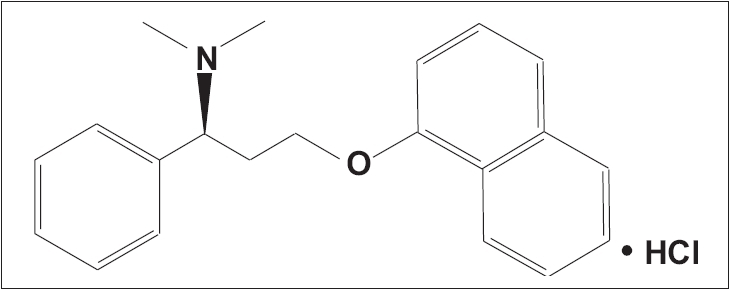
Molecular structure of dapoxetine: (+)−(S)−N, N-dimethyl-(α)-[2(1-naphthalenyloxy)ethyl]-benzenemethanamine hydrochloride

**Figure 6:**
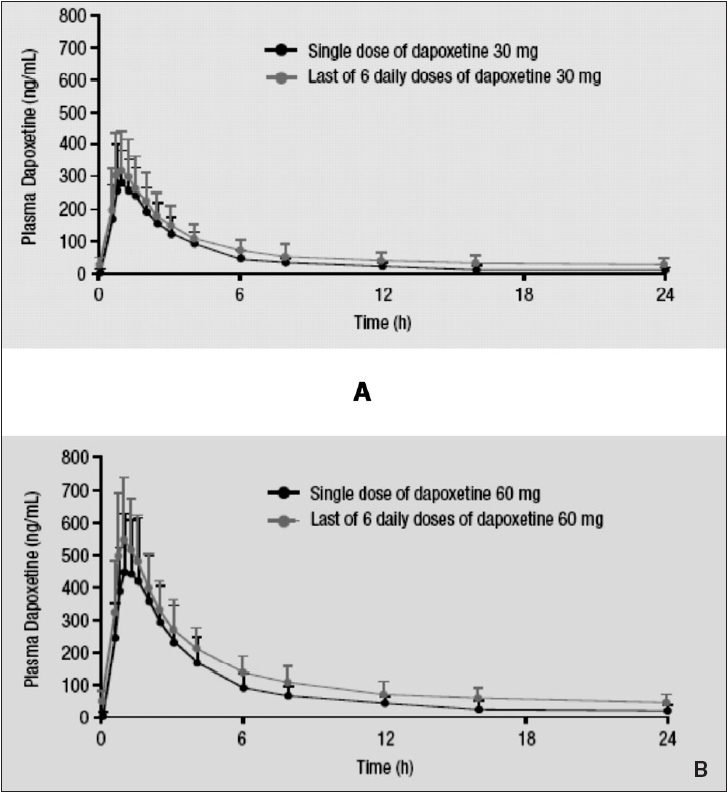
Plasma concentration profiles of dapoxetine after administration of a single or multiple doses of dapoxetine 30 mg (A) and dapoxetine 60 mg (B)[85]

Dapoxetine undergoes rapid absorption and elimination resulting in minimal accumulation and has dose-proportional pharmacokinetics, which are unaffected by multiple dosing [Figure 5]. The pharmacokinetic profile of dapoxetine suggests that it is a candidate for on-demand treatment of PE [Figure 6]. The pharmacokinetics of both single doses and multiple doses over 6-9 days (30, 60, 100, 140 or 160 mg) of dapoxetine have been evaluated. Dapoxetine has a T_max_ of 1.4-2.0 hours and rapidly achieves peak plasma concentration (C_max_) following oral administration.[84] Both plasma concentration and area under the curve (AUC) are dose-dependent up to 100 mg. The mean half-life of dapoxetine after a single dose is 0.5-0.8 hours and plasma concentrations rapidly decline to about 5% of C_max_ at 24 hours. The pharmacokinetics of dapoxetine and its metabolites were not affected by repeated daily dosing and steady state plasma concentrations were reached within 4 days, with only modest accumulation of dapoxetine (approximately 1.5-fold).[85] Food does not have a clinically significant effect on dapoxetine pharmacokinetics.[86]

No drug-drug interactions associated with dapoxetine have been reported. Coadministration of dapoxetine with ethanol did not produce significant changes in the pharmacokineticsof Dapoxetine and its metabolites.[87] Drug interaction studies demonstrate that tadalafil, a phosphodiesterase-5 inhibitor used in the treatment of ED, did not affect the pharmacokinetics of dapoxetine, whereas sildenafil increased the dapoxetine AUC by 22%.[88] However, this was not regarded as clinically important. Dapoxetine did not appear to affect the pharmacokinetics of tadalafil or sildenafil.

**Figure 7:**
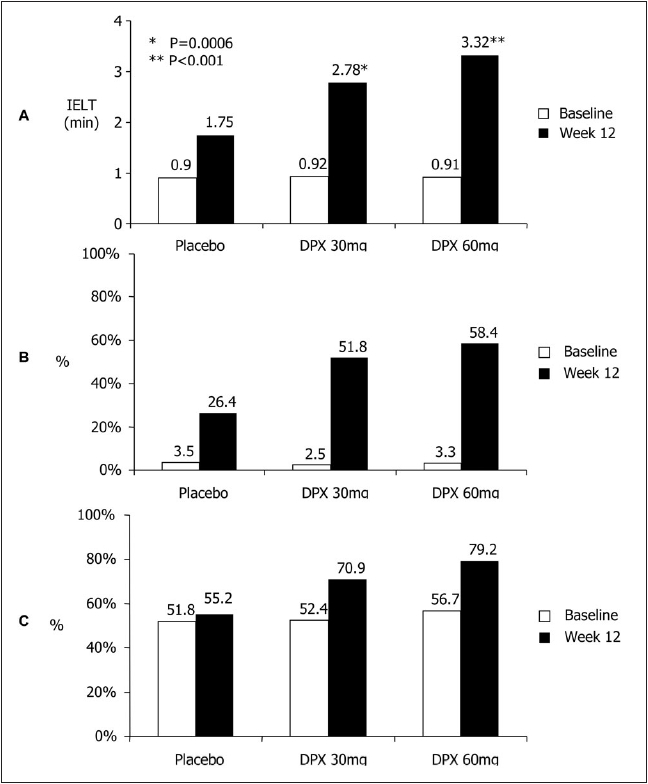
A. Increase in intravaginal ejaculatory latency time (IELT) from 0.9 minutes at baseline to 2.78 and 3.32 minutes at the end of the studywith dapoxetine 30 and 60 mg respectively. B. % of subjects rating control-overejaculation as fair, good or very good increased from 3.5% at baseline to 51.8% and 58.4% at the end of the study with dapoxetine 30 and 60 mg respectively. C. % of subjects rating Sexual Satisfaction as fair, good or very good increased from 51.8% at baseline to 70.9% and 79.2% with dapoxetine 30 mg and 60 mg respectively. (rating scale 0-5 scale, 0=very poor and 5=very good)[129]

Preliminary data suggest that dapoxetine (Johnson and Johnson) administered 1-2 hours prior to planned intercourse, is effective and well tolerated, superior to placebo and increases IELT 2-3 fold over baseline in a dose-dependent fashion [Figure 7].[89] In randomized, double-blind, placebo-controlled, multicenter, phase III, 12 week clinical trials involving 2614 men with a mean baseline IELT ≤ 2 minutes, dapoxetine 30 mg or 60 mg was more effective than placebo for all study endpoints.[90] IELT increased from 0.9 minutes at baseline to 2.78 and 3.32 minutes at the end of the study with dapoxetine 30 and 60 mg respectively. Mean patient rating of control-over-ejaculation as fair, good or very good increased from 3.5% at baseline to 51.8 and 58.4% at the end of the study with dapoxetine 30 and 60 mg respectively. Treatment-related side effects were uncommon, dose-dependent, included nausea, diarrhoea, headache, dizziness and were responsible for study discontinuation in 4% (30 mg) and 10% (60 mg) of subjects.

### On-demand treatment with tramadol

The efficacy of on-demand tramadol in the treatment of PE was recently reported.[91] Tramadol is a centrally acting synthetic opioid analgesic with an unclear mode of action which is thought to include binding of the parent compound and M1 metabolite to μ-opioid receptors and weak inhibition of reuptake of norepinephrine and serotonin.[92] Serotonin syndrome has been reported as an adverse effect of tramadol alone or in combination with SSRI class drugs.[93,94] In this double-blind, placebo-controlled study, the on-demand use of 50 mg tramadol, taken 2 hours prior to intercourse, exerted a clinically relevant ejaculation delay in men with PE with a 12.7 fold increase in IELT.[91] Additional flexible dose studies and long term follow-up studies to evaluate the risk of opioid addiction are required.

### Anaesthetic topical ointments

The use of topical local anaesthetics such as lignocaine and / or prilocaine as a cream, gel or spray is well established and is moderately effective in retarding ejaculation. A recent study reported that a metered-dose aerosol spray containing a eutectic mixture of lidocaine and prilocaine produced a 2.4 fold increase in baseline IELT and significant improvements in ejaculatory control and the sexual quality-of-life of both patients and their partners.[95] They may be associated with significant penile hypoanaesthesia and possible transvaginal absorption, resulting in vaginal numbness and resultant female anorgasmia unless a condom is use.[96–98]

### Phosphodiesterase inhibitors

Medications that inhibit the phosphodiesterase type-5 isoenzyme (PDE-5) such as sildenafil, tadalafil and vardenafil, are effective treatments for ED. Several authors have reported their experience with PDE-5 inhibitors alone or in combination with SSRIs as a treatment for PE.[99–112] The putative role of PDE-5 inhibitors as a treatment for PE is based upon the role of the NO / cGMP transduction system as a central and peripheral mediator of inhibitory nonadrenergic, noncholinergic, nitrergic neurotransmission in the urogenital system.[113] Several studies suggest that elevation of extracellular nitric oxide (NO) in the MPOA accelerates dopamine release and facilitates male copulatory behaviour of rats, whereas a decrease of NO reduces their copulatory behaviour.[114–116] Hull *et al* demonstrated that microinjection of the NO synthase inhibitor, N nitro L arginine methyl ester (NAME) not only decreased the number of erections but also increased the number of seminal emissions and decreased the latency to the first seminal emission.[117] The results indicate that not only does nitric oxide promote erection in intact male rats but it may also inhibit seminal emission.

Nitric oxide synthase isoenzymes are present in human seminal vesicle smooth muscle.[118] Several authors have reported the effects of NO donor drugs on electrically induced contractions and on tissue levels of cyclic guanosine monophosphate (cGMP) and cyclic adenosine monophosphate (cAMP) of isolated human seminal vesicle smooth muscle preparations. These authors have concluded that NO might be involved in the control of secretory activity and smooth muscle function of human seminal vesicles.[119–121] Consistent with this notion, Kriegsfeld reported that mice homozygous for *eNOS* gene deletion have striking ejaculatory anomalies.[122] A significantly higher percentage of mice with *eNOS* gene deletion as compared to normal controls, ejaculated during the testing period, requiring less stimulation and fewer mounts and intromissions.

A recent systematic review of 14 studies published in peer reviewed journals or the proceedings of major international and regional scientific meetings on the PDE-5i treatment of premature ejaculation, examined the role of NO as a neurotransmitter. This role was examined in central and peripheral control of ejaculation, the methodology of phosphodiesterase type 5 inhibitor (PDE-5i) treatment studies for PE, the adherence of methodology to the contemporary consensus of ideal PE drug trial design, the impact of methodology on treatment outcomes and the role of PDE-5i drugs in the treatment of PE.[123] These studies comprise a total of 1102 subjects suffering from PE and treated with sildenafil,[99,103–106,112] tadalafil[108] or vardenafil[107] either as monotherapy or in combination with SSRI drugs,[99,100,109,99–101,106–109,111,124] clomipramine[99] or topical anaesthetics.[109,112]

Most of these studies support a role for PDE-5i in the treatment of PE and speculate multiple mechanisms for their efficacy. These include 1) a central effect involving increased NO and reduced sympathetic tone, 2) smooth muscle dilatation of the vas deferens and seminal vesicles and 3) reduced performance anxiety. The smooth muscle dilatation of the vas deferens and seminal vesicles may oppose the sympathetic vasoconstriction and delay ejaculation. Better erections and downregulation of the erectile threshold to a lower level of arousal resulting in the requirement of increased levels of arousal to achieve the ejaculation threshold ultimately result in reduced performance anxiety.

The small number of publications and the lack of sufficient data preclude any meta-analysis of results. However, examination of the methodology of these studies, the adherence of methodology to the contemporary consensus of ideal clinical trial design[68] and the impact of study methodology on treatment outcomes, fails to provide any robust empirical evidence to support a role of PDE-5 inhibitors in the treatment of PE with the exception of men with PE and comorbid ED. Of the 14 studies reviewed, only one fulfilled these criteria and this study failed to confirm any significant treatment effect on IELT.[104]

Caution should be exercised in interpreting PDE-5i and on-demand SSRI treatment data in inadequately designed studies and their results must be regarded as unreliable. The extremely broad range of IELT fold-increases reported with sildenafil (2.7-15.0, mean 6.6), combined sildenafil and on-demand sertraline (3.3-10.0, mean 6.9) and combined sildenafil and on-demand paroxetine (6.6-14.9, mean 10.7) in this systematic review, is proof of the unreliability of inadequate study design. In contrast to these findings, the range of placebo IELT fold-increases was relatively narrow (IELT-range 1.2-1.6, mean 1.4) and was identical with the mean 1.4 IELT fold-increase reported in a meta-analysis of other PE drug studies.[125]

## PE AND COMORBID ED

There is evidence to suggest that PDE-5is alone or in combination with a SSRI may have a role in the management of acquired PE in men with comorbid ED. This systematic review includes 3 studies[105,107,111] of patients with PE with comorbid ED treated with a PDE-5i alone or in combination of sertraline. In 45 men with PE and comorbid ED treated with flexible doses of sildenafil (50-100 mg) for periods of 1-3 months, Li *et al* reported improved EF in 40 men (89%) and reduced severity of PE in 27 men (60%).[105] Improved EF was reported by all of the 27 men with reduced severity of PE, of whom 81.5% described themselves as satisfied or very satisfied. Contrary to these findings, only one of the 18 men (5.6%) who did not obtain improvement of PE reported treatment satisfaction.

In a group of 37 men with primary or acquired PE and a baseline IIEF EF domain score of 20.9 consistent with mild ED, Sommer *et al* reported and a 9.7 fold IELT increase and normalisation of EF (IIEF EF 26.9) with vardenafil treatment as opposed to only 4.4 fold IELT increase with on-demand sertraline.[107]

The high level of correlation between improved EF with sildenafil and reduced severity of PE reported by Li[105] and the superior IELT fold-increase observed with vardenafil reported by Sommer *et al* indicates that decrease in PE severity due to PDE-5i is due to improved EF.[107] The 4.4 IELT fold-increase observed by Sommer *et al* with on-demand sertraline is less than that reported in reviewed studies on men with normal EF (mean 5.57, range 3.0-8.5).[99,101,106,111] This suggests that men with PE and comorbid ED are less responsive to on-demand SSRIs and are best managed with a PDE-5i alone or in combination with an SSRI. Furthermore, Chia's report showed that addition of sertraline to the sildenafil treatment of men with ED and comorbid PE was associated with a lower IELT fold-increase (3.3). This in turn resulted in lower levels of treatment satisfaction as compared to men with lifelong PE and normal EF treated with on-demand sertraline, which suggests that this group of men are less responsive to pharmacotherapy.[111]

Thus, there are he advantages of PDE-5is as monotherapy or in combination with an SSRI in the treatment of acquired PE in men with comorbid ED. These advantages include 1) the ability to maintain an erection following ejaculation, 2) reduction of the erectile refractory period[104,126,127] and reliance upon a second and more controlled ejaculation during a subsequent episode of intercourse, 3) a reduction in performance anxiety due to better erections or downregulation of the erectile threshold. The downregulation of the erectile threshold to a lower level of arousal leads to the requirement for increased levels of arousal to achieve the ejaculation threshold.

### Surgery

Several authors have reported the use of surgically induced penile hypoanaesthesia via selective dorsal nerve neurotomy or hyaluronic acid gel glans penis augmentation in the treatment of lifelong PE refractory to behavioural and / or pharmacological treatment.[128] The role of surgery in the management of PE remains unclear.

## THE OFFICE MANAGEMENT OF PREMATURE EJACULATION

**Figure 8:**
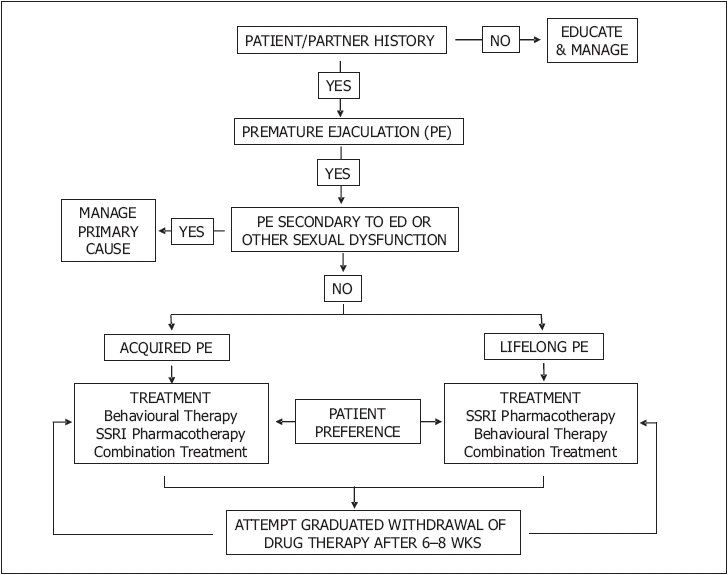
Algorithm for the office management of premature ejaculation[8]

Men with PE should be evaluated with a detailed medical and sexual history, a physical examination and appropriate investigations to establish the true presenting complaint and identify obvious biological causes such as genital or lower urinary tract infection [Figure 8].

Men with PE secondary to ED, other sexual dysfunction or genitourinary infection should receive appropriate etiology-specific treatment. Men with lifelong PE should be initially managed with pharmacotherapy. Men with significant contributing psychogenic or relationship factors may benefit from concomitant behavioural therapy. Men with PE secondary to ED can be treated with either ED-specific pharmacotherapy, e.g., PDE-5 inhibitors as monotherapy or in combination with PE-specific pharmacotherapy, e.g., daily or on-demand SSRIs. Recurrence of PE is highly likely following withdrawal of treatment. Men with acquired PE can be treated with pharmacotherapy and / or behavioural therapy according to patient / partner preference. Restoration of ejaculatory control in men with acquired PE is likely to occur following completion of treatment but is the exception in men with lifelong PE. Behavioural therapy may augment pharmacotherapy to enhance relapse prevention.

## CONCLUSION

PE is a common male sexual disorder. Recent normative data suggests that men with an IELT of less than 1 minute have “definite” PE, while men with IELTs between 1-1.5 minutes have “probable” PE. Although there is insufficient empirical evidence to identify the etiology of PE, there is limited correlational evidence to suggest that men with PE have high levels of sexual anxiety and altered sensitivity of central 5-HT receptors. Pharmacological modulation of the ejaculatory threshold using daily or on-demand selective SSRIs is well tolerated and offers patients a high likelihood of achieving improved ejaculatory control within a few days of initiating treatment, consequential improvements in sexual desire and other sexual domains. Daily administration of an SSRI is associated with superior fold increases in IELT compared to on-demand administration of SSRIs including dapoxetine. This is due to greatly enhanced 5-HT neurotransmission resulting from several adaptive processes which may include presynaptic 5-HT1a and 5-HT1b / 1d receptor desensitisation. It however, fails to directly address causal psychological or relationship factors and data are either lacking or scarce on the efficacy of combined psychosexual counselling and pharmacological treatment and the maintenance of improved ejaculatory control after drug withdrawal.
